# Exploring lessons from Covid‐19 for the role of the voluntary sector in integrated care systems

**DOI:** 10.1111/hsc.14062

**Published:** 2022-10-03

**Authors:** Juliet Carpenter, Ben Spencer, Tatiana Moreira da Souza, Youngha Cho, Jo Brett

**Affiliations:** ^1^ University of Oxford Oxford UK; ^2^ Oxford Brookes University Oxford UK; ^3^ University of Liverpool Liverpool UK

**Keywords:** community and voluntary sector, Covid‐19, integrated care systems, neighbourhood, Oxfordshire, qualitative research, VCSE

## Abstract

Integrated care systems (ICS) in England are partnerships between different health and social care organisations, to co‐ordinate care and therefore provide more effective health and social care provision. The objective of this article is to explore the role of the ‘Voluntary, Community and Social Enterprise’ (VCSE) sector in integrated care systems. In particular, the paper aims to examine recent experiences of the voluntary sector in responding to the Covid‐19 pandemic, and the lessons that can be learnt for integrated care provision. The article focuses on the case of Oxfordshire (UK), using a mixed methods approach that included a series of semi‐structured interviews with key informants in health and the VCSE sector as well as online surveys of GPs and organisations in the VCSE sector. These were complemented by two contrasting geographical case studies of community responses to Covid‐19 (one urban, one rural). Data were collected between April and June 2021. Interviewees were recruited through professional and community networks and snowball sampling, with a total of 30 semi‐structured interviews being completed. Survey participants were recruited through sector‐specific networks and the research arm of doctors.net.uk, with a total of 57 survey respondents in all. The research demonstrated the critical role of social prescribing link workers and locality officers in forging connections between the health and VCSE sectors at the hyper‐local level, particularly in the urban case study. In the rural case study, the potential role of the Parish Council in bringing the two sectors together was highlighted, to support community health and well‐being through stronger integrated working between the two sectors. The article concludes that enhanced connections between health and the VCSE sector will strengthen the outcomes of ICS.

**What is known about the topic**

- Integrated care systems (ICS) in England are designed to provide more effective health and social care provision at different spatial scales.
- Currently, the voluntary, community and social enterprise sectors (VCSE) are not integrated effectively into ICS.

**What this paper adds**

- Social prescribing link workers, together with locality officers, play a critical role in bridging knowledge gaps between the two sectors of health and the voluntary sector, particularly in an urban context. Stronger links between the two can support health and well‐being in the community.
- Outside urban areas, the Parish Council is well placed to play the role as intermediary between health and the voluntary sector in a rural setting.

## 1 INTRODUCTION

Integrated care systems (ICS) are a long‐term aim of the National Health Service (NHS) (Department of Health and Social Care, 2021; NHS, 2019). Since the introduction of the 2022 Health and Care Act, ICSs have been formalised as statutory bodies within the NHS. ICS are partnerships between different stakeholders, including the NHS, local councils and the ‘Voluntary, Community and Social Enterprise’ (VCSE) sector working within the ICS boundary, to provide more effective health and social care to local communities (Charles et al., 2018). A total of 42 ICSs have been introduced in England, organised through integrated care boards and integrated care partnerships. NHS Trusts are also joining together, to form ‘Provider Collaboratives’, new partnerships bringing together different aspects of health and social care, such as hospitals, mental health services and community services. ICSs are made up of a number of local authority areas, but a key characteristic of ICS policy is that commissioners and providers work over smaller geographies (at the so‐called ‘place’ level, such as the local authority), with teams delivering services within even smaller footprints (at the so‐called ‘neighbourhood’ level, such as the primary care network). One of the aims is to encourage closer working between different sectors, including health and the voluntary sector.

Recent research has highlighted some of the opportunities and challenges of closer integration between the health and VCSE sectors. The King's Fund notes that partnership working between health and the voluntary sector can contribute to people living longer in better health (King's Fund, 2021a), with impacts on reduced hospitalisation. However, the Institute for Voluntary Action Research (IVAR) has demonstrated the need for greater understanding, including a common language to talk about improvements to health and well‐being at the local level. They also emphasise the importance of co‐designed, integrated and asset‐based approaches to health and well‐being that provide locally relevant solutions, co‐designed between the health and VCSE sectors (IVAR, 2014; IVAR, 2016). Croft and Currie (2020) highlight the importance of VCSE involvement in delivering integrated care, through workforce capacity development and specific coordinating roles. However, they also warn against the potential for exploitation of VCSE organisations, whereby they become replacements for health and social care provision, rather than a complementary service within an integrated team.

**FIGURE 1:**
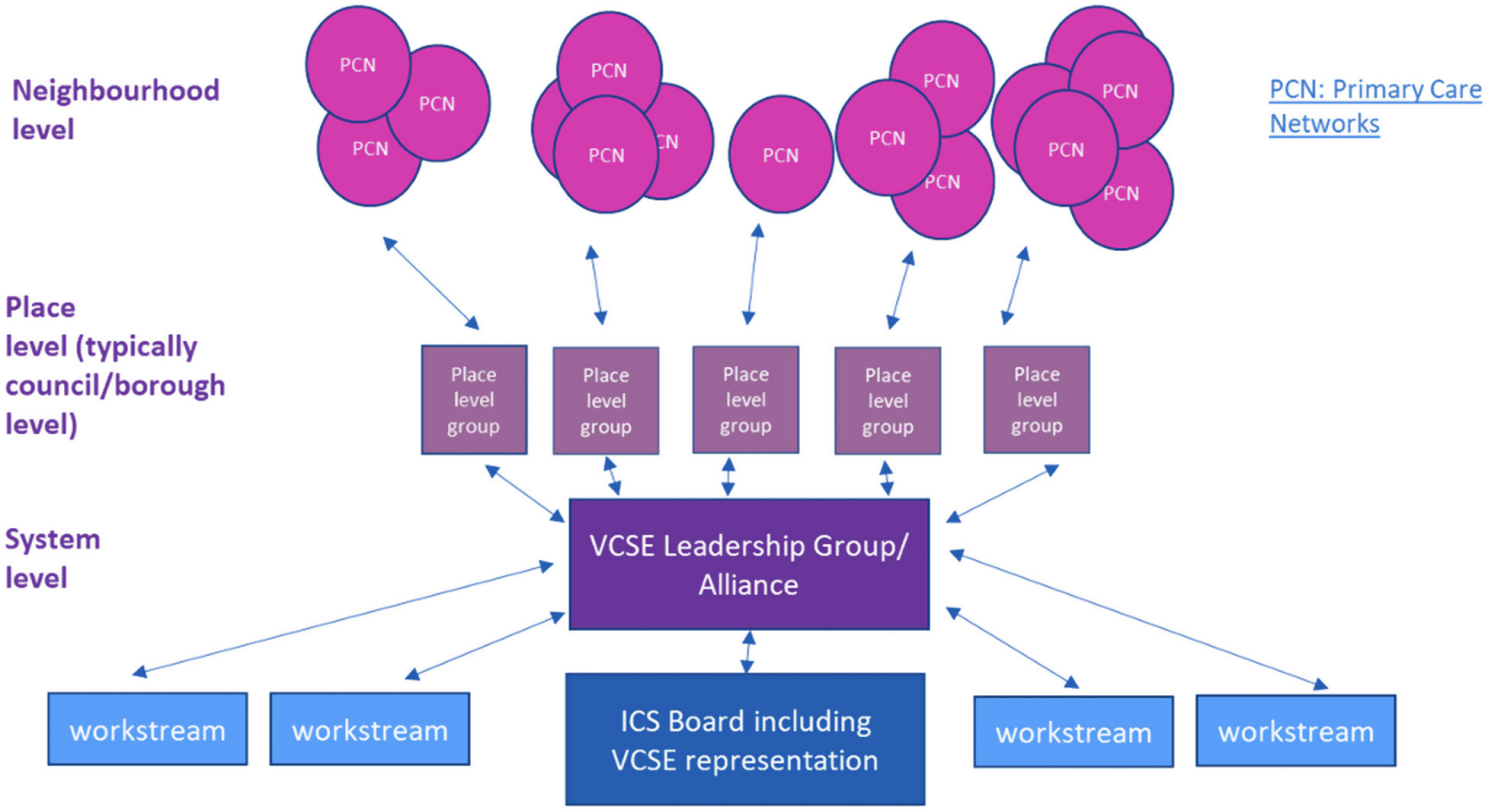
A good practice model of VCSE engagement at a system, place and neighbourhood level. Source: “Joined Up Care Derbyshire”. https://joinedupcarederbyshire.co.uk/get‐involved/voluntary‐community‐and‐social‐enterprise‐leadership‐programme

The need for strong and mature relationships in co‐production between the VCSE and health sectors is also highlighted by the King's Fund (2018). These themes were picked up by the NHS's VCSE Health and Wellbeing Programme, launched in April 2017, to promote co‐production in the creation of person‐centred, community‐based health and care, to support more effective and equal health outcomes. One strand of this work was the ‘Leadership Programme’, where funding and facilitation support were available to develop place‐based VCSE Alliances within ICSs. Building on research by the National Council for Voluntary Organisations (Pedro & Baylin, 2020), a series of VCSE Alliances have been funded, to establish networks of VCSE and health stakeholders in ICS areas. One such Alliance in Derbyshire has produced a good practice schema (Figure 1) of how the VCSE Alliance in their area can be embedded within the three different components of the ICS: system, place and neighbourhood. It illustrates the importance of integrating a network of VCSE voices at all three levels. However, there is currently a lack of research on how VCSE partners can work more effectively in partnership within the new ICS structures for better health outcomes (King's Fund, 2021b).

During the Covid‐19 pandemic, one of the key features of the societal response to the crisis has been the rise in mutual aid organisations and related activity through the VCSE sector, particularly in supporting vulnerable and older populations (NLGN, 2020). This has included assistance such as food shopping, prescription collection, dog walking and phone buddying/befriending. The Academic Health Science Network (AHSN) has carried out research on how lessons from the Covid‐19 pandemic can inform health and care systems of the future (AHSN (Academic Health Science Networks), 2021). Their recommendations include the need to build on existing relationships and form new partnerships; the importance of greater co‐production in multi‐stakeholder partnerships that include health sector professionals, patients and the public; and the necessity of understanding population needs to address inequalities.

Building on the AHSN's broad study, our objective in this research was to focus in detail on the case of Oxfordshire (UK), to investigate how the recent local experiences of VCSE organisations responding to Covid‐19 can be built upon, to strengthen integrated partnership working within the local ICS for Buckinghamshire, Oxfordshire and Berkshire West (BOB). The case of Oxfordshire was selected as it brought particular insights, given its mixed urban and rural settings. This offers contrasts between the two contexts, as well as having relevance to other areas with a similar geographic mix.

The overall aim of the research was to identify key components that have facilitated partnership working between the VCSE and health sectors and to provide recommendations for the ICS on how the two sectors can work together more effectively, to the benefit of population health and well‐being. The specific research questions addressed were: How have local communities responded to the pandemic through VCSE initiatives? How effective have these initiatives been in reaching older and more vulnerable people, and in addressing isolation, mental health concerns and well‐being? And what is the potential for policy learning from these experiences, to strengthen voluntary sector involvement in the local BOB ICS health system?

## 2 METHODS

### 2.1 Study design and setting

This was a mixed‐methods study, using primary and secondary sources to examine the role of VCSE organisations in the county of Oxfordshire (UK) in supporting older and more vulnerable people, and the potential for closer voluntary sector involvement in health provision within the BOB ICS (Buckinghamshire, Oxfordshire and Berkshire West). The methods included semi‐structured in‐depth interviews and online surveys at the regional level, complemented by two place‐based case studies, one urban and one rural, drawing on the experiences of three stakeholder groups: health providers, the voluntary sector and local residents.

The study was constructed using a three‐stage methodology. Building on an initial literature review, a series of in‐depth semi‐structured interviews was conducted in the first stage of the research, with key informants in both the health and VCSE sectors. A qualitative approach was used to gain an in‐depth understanding about experiences of, and responses to, the pandemic from different actor perspectives (VCSE, health and communities). A total of 15 people were interviewed virtually on Zoom in this first stage (13 from the VCSE sector, one from health and one in social prescribing, that combines VCSE and health perspectives), to explore their different experiences of the pandemic, and how health and the VCSE sector could be more closely integrated.

The second stage involved distributing two online surveys to GPs and the VCSE sector, focusing on cross‐sector collaboration and support to older and vulnerable groups during the pandemic. The GP surveys were circulated through the research arm of Doctors.net.uk (M3) focusing on Oxfordshire and the wider South East. The VCSE survey was distributed through the volunteer organisation ‘Oxfordshire Community and Voluntary Action’ (OCVA) via its members' newsletter. A copy of the survey for GPs can be found in Appendix S1, while a copy of the survey for voluntary and community groups can be found in Appendix S2.

The third stage involved case studies in two contrasting localities: Case A, a neighbourhood of Oxford, and Case B, a rural village in Oxfordshire. These two cases were selected in consultation with the project Advisory Board, as examples of strong community responses to the pandemic, to explore those elements that worked well, as well as aspects that worked less well, and the reasons why that might have been. The two areas were also interesting to explore, given their different socio‐economic profiles. Case A is a mixed‐tenure urban housing estate originally built in the 1940s as social housing and located on the edge of the Oxford city. According to the ‘Indices of Deprivation’ data for 2019, the neighbourhood falls within the most deprived quintile nationally (Oxford City Council, 2019). Case B, on the other hand, is a relatively well‐off rural village and civil parish of under 400 residents, located outside Oxford. The village has no amenities, shops or a pub, and residents are dependent on private transport, due to a very limited public transport service.

For the two case studies, a total of 15 semi‐structured interviews were carried out. In Case A, nine interviews were completed: three involving the VCSE sector, two in health, and four residents (1 who received support and 3 who provided support). In Case B, a total of six semi‐structured interviews were completed: three in the VCSE sector and three residents (all of whom received support). Interviews were carried out either by Zoom or telephone.

The table below (Table 1) summarises the characteristics of the interview participants, including the type of organisation that stakeholders were engaged with, to illustrate the spread of views included, from national and regional perspectives, through to local and hyper‐local levels.

**TABLE 1:** Characteristics of interview participants

	Number of interviewees
National organisations	1
Regional offices of national organisations	3
Regional/county level organisations	5
City level organisations	7
Local and hyper‐local organisations	7
Local residents	7
Total	30

The research was also supported by an Advisory Board with six members across the health, VCSE, local authority and academic sectors, who met virtually three times during the study in February, April and June 2021.

The project was granted ethical approval on March 18, 2021 by the University Research Ethics Committee at Oxford Brookes University, ethics registration number 201455. Oral consent was obtained prior to the online interviews, on Zoom or by telephone. Consent from survey participants was obtained using the survey tool.

### 2.2 Data collection

The researchers conducted the semi‐structured interviews and distributed the online survey over a 3‐month period, between April and June 2021. Due to the pandemic, all methods were designed to be online to avoid face‐to‐face interaction, using zoom, telephone and the online survey tool Qualtrics.

Semi‐structured interviews lasted approximately 60 min. The topic guides for the interviews were informed by the literature review, with advice from the Advisory Board. The three topic guides, one for each group (health, the voluntary sector and residents) were tailored to each specific category, but broadly covered their experiences during the pandemic, and the links between the health sector and community provision of support. In each case, respondents were asked to reflect on aspects of support that had worked well, those that had worked less well, and the lessons that could be drawn for future cross‐sector working.

The online surveys were designed to access a wider range of views, from both VCSE organisations, and from GPs about their experiences of working with the VCSE sector, before the pandemic, during the health crisis, and their views on how cross‐sector working could be enhanced in the future. The online survey was completed by a total of 50 GPs and seven VCSE organisations. A total of 12/50 of the GP surveys were completed from Oxfordshire, with a further 26/50 from elsewhere in the South East. The remaining 12/50 came from London, East and West Midlands and the East of England. All seven of the VCSE organisations were located in Oxfordshire.

### 2.3 Data analysis

All 30 semi‐structured interviews were recorded, transcribed by researchers and analysed using thematic analysis (Braun & Clarke, 2006). The coding frame was developed by three of the researchers (J.C., B.S., T.M.D.S.) who read and re‐read the transcripts and compared codes to ensure reliability and validity of the analysis. The coding frame was divided into three main codes: perspectives on how the health and VCSE sectors have worked together during the pandemic, lessons learnt for integrated working in the future and differences between responses from interviewees in the urban and rural case studies. Data from the online surveys were integrated into the interview analysis through descriptive statistics.

### 2.4 Terminology

There are a number of different ways of referring to the ‘community‐related’ sector, for example, ‘civil society’, ‘mutual aid organisations’, the ‘Third Sector’, the ‘Voluntary and Community Sector (VCS)’ and the ‘Voluntary, Community and Social Enterprise’ sector (VCSE). In this research, we have adopted the term VCSE, to include any organisation (incorporated or not) working in the ‘community‐related’ sector. Where possible, in light of the research data, we make the distinction between small community‐based neighbourhood groups, such as local street‐based WhatsApp groups that have sprung up during the pandemic, in contrast to large, registered charities, such as Age UK and Mind that operate locally, regionally, nationally or even internationally. In the research, we use the general term VCSE to cover these different types of organisation, although we recognise that there are significant differences between them, and that particular comments in this paper may not be relevant to all organisations covered by this term.

We also recognise that the ‘health sector’ and the 'VCSE sector' do not represent single voices, but many voices within each sector, and referring to these broad umbrella terms will inevitably involve grouping together a range of disparate cultures and perspectives. However, we felt that this would be the most effective approach to access a range of perspectives in a short period of time, without excluding certain voices in the broad sectors of health and VCSE. Within the ‘health sector’, we have included ‘social prescribing link workers’, who straddle both the health and VCSE sectors.

## 3 RESULTS

The results are divided into three main themes: Community responses to the pandemic; Effectiveness of community initiatives during the pandemic; and Potential for policy learning for ICSs. Points are illustrated with verbatim quotes from participants.

### 3.1 Responses to the pandemic

In line with experiences elsewhere (Dayson & Woodward, 2021; Ellis Paine et al., 2022; NLGN, 2020), a strong mobilisation of local community groups and volunteers was reported, in response to the pandemic. A common motivation for volunteers to come forward was wanting to support vulnerable groups in a time of crisis, in addition to the benefits that the social contact offered for the volunteers themselves. These were common motivations in both the rural and urban case study settings. In the urban setting of Case A, the local social infrastructure (Klinenberg, 2018) was already in place with long‐established and trusted community leaders based out of the local community centre, who spearheaded the mobilisation of volunteers in response to the pandemic. In the rural village of Case B, there was no formal infrastructure in place initially but individuals within the Parish Council took a leadership role in mobilising local residents to sign up for volunteering roles to support more vulnerable members of the community.

Therefore, the responses to the pandemic linking the health and VCSE sectors differed in the two case study areas. In the urban case study, there were already strong links between the GP practice and the local community. This relationship had been built up over a number of years through a partnership between the social prescrib link worker (Polley et al., 2017), and a dedicated locality officer on the ground, who promoted asset‐based community development (ABCD) (Kretzmann & McKnight, 1993, 1997).

The role of locality officers was seen in the urban Case A as key to developing locally relevant, bottom‐up services. One respondent commented that these locally embedded workers have a critical role in helping the community not only to define their own community priorities, but also to identify appropriate solutions within the community, and where the skills are available, to contribute to delivering those solutions.

While such locality officers have been highly effective in many cases during Covid‐19 in Oxfordshire, there have also been challenges in terms of the VCSE offer that was available for local social prescribing link workers:

One of the impacts of Covid has been that a lot of the things that used to exist, that you could ‘social prescribe’ to, just didn't exist anymore. [Social prescriber]

GPs responding to the survey also cited the challenges of reduced funding and dwindling resources for organisations delivering services that connect with social prescribing. It was noted that the volume of need has increased during the pandemic, at a time of reduced services. GPs also reported a lack of awareness of available social prescribing services and the difficulty of bringing organisations together during the pandemic. A summary of the survey results from GPs can be found in Appendix S3.

The ‘Locality Hubs’ set up by Oxford City Council in March 2020 as a response to the pandemic, and place‐based working more generally, were widely praised by interviewees, linking with an asset‐based approach to community development. In Case A, the Locality Response Hub included the local community association, a partnership of local Health Centres, social services, the police, a local church and other local VCSE organisations. Also involved was a Community Health Worker employed by Oxford City Council using Community Infrastructure Levy (CIL) funding raised through a local housing development The Hub held weekly coordination meetings to ensure community needs were being met, and there were direct links with the GP surgery through the health partnership.

In the rural area of Case B, on the other hand, the responses to the pandemic were centred around a more informal support network, that was initiated by two local women living in the village, one with close connections to the Parish Council. Just before the lockdown in March 2020, they took the initiative to leaflet all households in the village, asking people to come forward either to offer support through volunteering, or to self‐identify as potentially needing assistance in the event of a lockdown, due to vulnerability. This formed the basis of the official response strategy from the Parish Council, which led on a number of initiatives to support villagers, in particular vulnerable and elderly residents. This included purchasing and distributing PPE supplies, setting up a ‘buddy’ scheme where two volunteers were assigned to each household identified as vulnerable, and in particular, focusing on addressing potential isolation by ensuring vulnerable households were connected to the internet and had IT devices to access services and video‐calling applications. The aim was to reduce social isolation and thus address wider well‐being outcomes.

Since there were very few links between local health services and the village's community support networks, a system of prescription collection was established. This was an informal arrangement between the organisers of the village's pandemic response and two local pharmacists, with named volunteers assigned to collect particular prescriptions. There were no links with more formal NHS health services or any local social prescribers in relation to activities run by village residents. Such links were seen as potentially problematic, given the nature of more ad hoc or voluntary activities, which by their nature are not necessarily regular. Nonetheless, it was suggested that a more formal contact point within the village serving as a hub for signposting information about local initiatives and community assets that could potentially be relevant for social prescribing purposes would be useful, particularly for older residents.

### 3.2 Effectiveness of the pandemic responses

Survey responses from VCSE organisations indicated that the hyper‐local support offered to local vulnerable groups during the pandemic was seen as a vital initiative, particularly for older people who were at risk of social isolation during lockdown. Phone buddying allowed for safe contact, while delivering services such as shopping or food parcels to those self‐isolating was also seen as a valuable way of checking that vulnerable individuals had regular socially distanced contact. A summary of the survey results from the VCSE sector can be found in Appendix S4.

While community responses to the pandemic were largely seen as a significant success, it was recognised that the role of effective partnerships between the health and VCSE sectors was key to this. A very strong and recurring theme of the interviews in Case A was the time, resources and skills required to build understanding, trust and positive relationships between health and community groups through partnership:

That stuff takes time and it's easier to go it alone and it's harder and more resource intensive and everything else to do things in partnership. [Locality officer]

Others felt that this challenge mainly falls on the Primary Care Networks (PCNs), which are under pressure to build these partnerships alongside their other roles:

[There's] ‘a lot of onus on PCNs to set this stuff up and organize it, and they can't be all things to all people and they can't do commissioning or service design or partnership work all day long when they are GPs, so it does feel like a crucial part of the jigsaw is missing.’ [Social prescriber]

Investment in social prescribing link workers (Tierney et al., 2020) within PCNs was welcomed but some interviewees thought that more was required to maximise the benefits from this, including providing infrastructure to support their work, as well as networking and training opportunities for the link workers.

Through the survey, many GPs also reported on the benefits of working with the VCSE sector during the pandemic. In particular, they cited increased community cohesion at a time when close family, perhaps living at a distance, were unable to help vulnerable members of the local community. Volunteers became a point of referral and support, helping people with both practical and social aspects of need. The most common benefits reported were support for the isolated elderly, support for mental health and supporting the vaccination centres. GPs felt volunteers were enthusiastic, flexible, helpful and good team players. They felt that working collaboratively improved communication and helped relieve the pressure from GPs, at such a busy time.

However, working with the voluntary sector was also reported to be challenging. As well as reduced funding and staffing of voluntary groups, and the loss of face‐to‐face interactions, there was also concern around confidentiality and volunteer safety checks. One GP referred to the challenge as ‘crossing boundaries’, between the health and VCSE sectors. Others reported concerns around the level of knowledge that the volunteers had, and the perceived lack of training of volunteers with appropriate skills. Furthermore, some GPs reported that they were unsure which voluntary organisations to contact, and felt the voluntary organisations did not promote awareness of their services effectively within healthcare services. Over 70% of those GPs who responded to the survey linked with just one VCSE organisation, with a further 18% only linking to two organisations. This suggests the need for more comprehensive signposting of local VCSE organisations and the services that they provide that could be useful for social prescribing.

### 3.3 Potential for policy learning on linking the VCSE and health sectors

At the neighbourhood PCN level, drawing on the responses from the GP survey, four key elements were identified as necessary for a closer integration of health with the VCSE sector. These were information and communication (with GPs needing to be aware of what is available); facilitating processes for connection (including stronger links between local VCSE organisations and social prescribing link workers); the issue of funding, in particular securing funding for smaller VCSE organisations to provide stability; and ‘patient willingness’, with some GPs identifying a reticence on the part of patients to embrace social prescribing as a valid response to their health concerns.

At the wider place and system levels, there was a recognition that if the VCSE sector is going to take a role in ICSs, the issue of partnership working needs to be addressed, in particular, to ensure the involvement of grassroots organisations at the hyper‐local level, which might otherwise be neglected in favour of larger and more established organisations.

This lack of engagement with grassroots organisations is due to a range of issues that lie at the heart of the challenges of building a diverse partnership between the two sectors. First, it relates to the opportunities that are open to different locally based organisations, to play a role in partnerships, which are often limited due to an organisation's lack of capacity. There are also issues around support and funding for partnerships, which excludes those partners without the resources to get involved. Barriers also exist which prevent working beyond organisational boundaries, which can be difficult to overcome. Finally, and critically, there is a need to recognise and overcome power imbalances between different partners, to address some of the fundamental obstacles to involving grassroots organisations.

An important lesson in terms of Integrated Care Systems is how they commission and create the environment and the conditions for partnership working. [Health stakeholder]

Covid has created a lot of opportunities really, because of the fact that organizations have worked really collaboratively, […] ‐, and now the challenge for ICSs is how to harness that and resources, and support it for the longer term. [VCSE representative]

Concerns about integrating the VCSE sector into ICSs were reflected by one interviewee:

I do hope that the ICS doesn't sit as a hierarchical organization at a very senior policy level, but it does do what in theory it is truly meant to be doing, engaging across sectors and is truly representative, primarily of the communities and individuals themselves. [VCSE representative]

One potential solution that was suggested involves commissioning based on different reporting and funding models, such as Participatory Grantmaking, to fund and support hyper‐local groups to develop appropriate services. This implies a culture shift within the ICS, and the need to share power and decision‐making:

From deciding on priorities together, and then designing and developing solutions together, […] it's a big cultural shift, and it's about the sharing of power. [Health stakeholder]

Working at a local and hyper‐local scale was seen as a particular challenge for ICSs given their large geographical size.

BOB is like a huge area […] to be doing this, and there's a real risk that we lose the ability to work in partnership and to do things in the community development, community capacity‐building way, and everything gets reduced to bigger service providers which loses so much. [VCSE representative]

A number of respondents referred to the nascent BOB‐wide VCSE Alliance. This is an initiative run through the VCSE Leadership Programme, with membership open to any VCSE organisation across BOB that signs up to Alliance principles (e.g. collaboration and transparency). It was seen as a positive development since it will provide representation and advocacy for the VCSE sector within the ICS and BOB workstreams.

Obviously, as part of the integration process […] we can actually be starting to develop priorities, we're all around the table together, working on the priorities together, and then talking about what the shared targets can be and how we can achieve those targets as a group of commissioners, providers and planners, and people hopefully, at some point… It's about setting up, establishing and embedding the systems across BOB, that bring the VCSE as equal partners to the table, to the ICS. [Health stakeholder]

These moves to engage more fully and transparently with the VCSE sector were welcomed by respondents. But as many interviewees recognised, it will take time to put systems and structures in place, to embed the VCSE sector within BOB, and to shift mindsets to embrace the VCSE sector in decision‐making around the table.

## 4 DISCUSSION

The research demonstrates, through stakeholder interviews, online surveys and the two case studies, that the pandemic mobilised different organisations and local volunteers to support vulnerable community members in their neighbourhoods. In both areas, the pandemic responses were seen as effective in supporting well‐being through volunteer engagement. However, there were differences between the two cases. In urban Case A, this led to a strengthening of the existing ties between the health and VCSE sectors in the area and demonstrated the key role of social prescribers in forging links between health and the VCSE sectors at the neighbourhood level, with clear policy implications for the ICS agenda to support local integrated approaches to health and well‐being. In rural Case B, community mobilisation was not connected to local health services due to the village's relative isolation, the physical distance to GP practices and absence of local formal health and well‐being services. There was an absence of contact between the VCSE sector and health agencies and sectors, which led to limited health‐related support in the rural setting.

The NHS Long‐Term Plan set a target that by 2023/2024, every GP practice in England will have access to a social prescribing link worker and by then, a target of 900,000 people has been set for those referred by a social prescriber (King's Fund, 2020). Social prescribing is already embedded in health practice (Costa et al., 2021; Pescheny et al., 2020), and is seen as being key to the delivery of an integrated health service in the future (Bickerdike et al., 2017; Drinkwater et al., 2019). The urban Case A supports the case for a strong role for social prescribers in linking health and the VCSE sectors. However, one of the challenges relating to social prescribing is the volatility of the availability of local services and activities to which to refer patients. The fleeting nature of some activities and initiatives means that social prescribers have to be continuously up‐to‐date with what is offered locally. This is directly linked to the ability of these small organisations to secure and maintain funding, as well as their access to volunteers and specialised personnel. Funding cuts in recent years and the current financial climate have created volatility and undermined the ability of the sector to meet local needs, which is destabilising for social prescribers. However, as Knapp et al. (2013) have demonstrated, there can be significant additional financial benefits to the health service from adequate funding of community‐capital‐building initiatives. Similarly, Parsfield et al. (2015) have demonstrated the social value of investing in communities, including the voluntary and community sector, by connecting people to one another in their local areas, with the potential for positive impacts on health and well‐being. Adequate core funding is crucial to the future of the VCSE sector and its role in the ICS, to ensure more stable and long‐lasting local activities and programmes, particularly in the light of possible future austerity measures due to the pandemic.

As a result of this instability, social prescribers very often link to more formal or substantial VCSE groups that are well‐known and well‐established in an area. This is due to the challenges of working with smaller providers related to the sustainability of opportunities, as well as those associated with whether the true value of small providers is fully understood by commissioners (Dayson & Batty, 2020). The research showed that in many cases, the more informal community‐based networks and support, that are so vital to neighbourhood cohesion, are ‘off the radar’ in relation to social prescribing. Dayson and Batty (2020) also raise this issue, highlighting the importance of ensuring the ongoing existence of a healthy and thriving ecosystem of small and hyper‐local community providers in a locality, for social prescribing. Connections between the health sector and these hyper‐local community resources would strengthen the potential for locally‐based community initiatives to impact on residents' health and well‐being. Engaging in the mapping of VCSE provision, combined with local on‐the‐ground knowledge from individuals in key umbrella organisations, is an initiative that would go some way to addressing this issue, and would be relevant to both urban and rural areas.

However, referring patients to local VCSE organisations also brings challenges to the health sector in relation to quality standards, health and safety issues, background checks of volunteers and personnel (e.g. the UK's Disclosure and Barring Service), as well as confidentiality issues related to patient health records. There are also issues around evaluating the evidence of the effectiveness of social prescribing interventions, and how conventional measures do not necessarily capture the benefits of such interventions (Bickerdike et al., 2017). These issues can represent very real barriers to closer integration of the VCSE and health sectors. It is important that these critical stumbling blocks are addressed more broadly and resolved in the context of the ICS, as they could represent significant obstacles to deeper engagement between the two sectors. Some of these issues could be addressed by establishing training and safe‐guarding protocols which would reassure commissioners on safety issues. Regarding monitoring and evaluation, a greater understanding and awareness among commissioners of non‐conventional ways of measuring benefits would contribute to recognising the non‐quantifiable outcomes and impacts of community‐based interventions.

Complementing the role of a social prescriber at a local level in an urban setting, the research demonstrated that one of the keys to successfully linking health needs and VCSE activity is the existence of a ‘Community Worker’ or 'Locality Officer' embedded within a neighbourhood. These community‐based roles are critical to building understanding, trust and positive relationships locally, given their reach into the community. They act as an intermediary or ‘bridge’ between different organisations and sectors, with an overview of hyper‐local needs and potential VCSE provision in the locality. In Case A, it was clear from interviews that the role of locality officer was critical in linking the community with services which contribute to physical and mental well‐being, and contributed to thinking about a more holistic approach to health and well‐being. There is a strong case to be made for similar roles to be well‐supported and well‐funded, with adequate training and support infrastructure in place to ensure successful recruitment and retention of these key positions.

In a rural setting, Parish Councils have the potential to play this pivotal intermediary role between local VCSE provision and the health sector. A nominated Councillor would be well‐placed to have an overview of local provision, and could sign‐post enquiries from the health sector to appropriate local clubs and activities. Defining this role at the level of the Parish Council brings stability, embedded within the pyramid structure of local government. This arrangement would avoid the risks inherent in more informal networks that currently exist. These current challenges include the precarity of relying on a small number of individuals to keep a network going, as well as the potential for conflict due to personal relations leading to exclusion. There are also safeguarding risks that would be lessened in a more formal structure.

However, such an arrangement would be dependent on a number of factors, including whether the Parish Councils would be willing and able to take on this role. Local parish politics can be fraught with tensions, and there are also questions about adequate capacity and appropriate training in community development work. Nevertheless, in certain circumstances, such a brokerage role could potentially facilitate the links between community sector opportunities and the health sector in a Parish Council context.

At the ICS ‘macro’ system level, for a more effective engagement between the VCSE and health sectors, it is clear from the study that a broad engagement with VCSE voices, both large and small, is needed to ensure integration within ICS structures. Initiatives such as the VCSE Alliance are working towards such a goal, although it is recognised that this will involve a significant culture shift within the constituent parts of the ICS. One component of this is ensuring that the VCSE sector has adequate representation on the Senior Leadership Group, as well as within its main work programmes and workstreams. These VCSE members would be representing the interests of the broader sector, rather than their individual organisations.

The value of linking health and the VCSE sector, for positive health outcomes has been demonstrated internationally, drawing on experiences of social participation interventions in the community that bring benefits in terms of addressing social isolation. For example, Saito et al. (2019) show how Japan's system of *ikoino saron*, or ‘gathering salons’ for people over 65 years have contributed to the government's long‐term care prevention plans. The salons, managed by local volunteers and with low participation fees, are held once or twice a month in local community spaces, where participants can meet, exchange ideas, and take part in activities. In 2017, over 85% of Japanese municipalities had implemented the salons in their localities. Studies have shown that participation in these salons is associated with a halved incidence in long‐term care needs, and around a third reduction in the risk of dementia onset. Lessons from this integrated approach could usefully inform how the VCSE and health sectors in England could work more closely together to fulfil the aims of ICSs going forward, with a shift towards prevention and self‐care.

### 4.1 Strengths and limitations

One of the key strengths of this study is the inter‐sectoral focus, exploring the links between the health and community sectors. Further added value was provided through drawing lessons from the pandemic, which provided a unique context to study these critical linkages. However, the research also had a number of limitations. Restricting the study to Oxfordshire meant that it was limited in its scope, although the urban and rural cases provided interesting contrasts.

Although the survey link was sent to a range of different organisations to cascade through their networks, the response rate was limited and non‐representative. Similarly, the number of participants interviewed was limited, with only one each from the health and social prescribing sectors, despite approaches to a wide group of potential interviewees. This reflects the severe pressures that the health service is currently under, more broadly due to underfunding, but in particular recently due to the pandemic. Additional research would endeavour to engage with a wider group of respondents.

The research also focused primarily on health and the voluntary and community sectors, rather than social care. Therefore, it did not address issues of the gap between health and social care, joining up these two dimensions, or other issues related to local authority involvement and their contribution to health and well‐being. These issues would benefit from further research.

## 5 CONCLUSIONS

Closer working with partners in the VCSE sector will be crucial for integrated working in the newly created ICSs. The VCSE sector is uniquely placed to provide a link between health services and local communities, to support population health. The research revealed a number of opportunities and barriers to joint working between the health and VCSE sectors which were highlighted by the experiences during the pandemic. In relation to opportunities, the pandemic brought different organisations together in crisis mode, and relationship building that can generally be time‐consuming and complex, materialised relatively quickly and with minimal friction. New partnerships formed that can be built upon in the future.

However, there were also a number of barriers to joint working, including different cultures and mindsets in the health and VCSE sectors, leading to a lack of understanding between the two groups, and hindered by their different languages. It was also evident that financial constraints in the VCSE sector, both now and particularly in the future, will limit the capacity of VCSE organisations to reach out beyond their core mission, to invest in collaborative work in ICSs. These are all issues that need to be considered when strategising around the partnership working with the voluntary sector in ICS in the future. Future research could be usefully focused on how to address the barriers to closer integration, in particular exploring the most effective way of forging partnerships with local and hyper‐local grassroots organisations, through the intermediary of locality officers and social prescribing link workers.

## FUNDING INFORMATION

This research has been funded by the Healthy Ageing and Care Network at Oxford Brookes University.

## CONFLICT OF INTEREST

The authors declare that they have no known competing financial interests or personal relationships that could have appeared to influence the work reported in this paper.

## Supporting information


Appendix S1‐S4

## Data Availability

The data that support the findings of this study are available from the corresponding author upon reasonable request.
