# Supplementary material for: Exploring lessons from Covid‐19 for the role of the voluntary sector in integrated care systems
Source: Health Soc Care Community. 2022 Oct 3;30(6):e6689–98. doi: 10.1111/hsc.14062 (PMC9874554; doi:10.1111/hsc.14062)
Supplement: Supplementary file 1 — Appendix S1‐S4 [file HSC-30-e6689-s001.docx]

**Appendix 1 – Survey for GPs**

Exploring lessons from COVID for the role of the voluntary sector in Integrated Care Systems

**Q1**

What year did you qualify as a GP?

**Q2**

How long have you worked in your current location?

| Year(s) | ______________________________ |
| --- | --- |
| Month(s) | ______________________________ |

**Q3**

What is the postcode of your practice?

Please enter first half of postcode only

|  |
| --- |

**Q4**

How many GPs (FTE) are in your practice?

|  |
| --- |

**Q5**

Before the COVID pandemic, what contact did you have with informal local community groups in providing health and/or social care?

- A lot (1)
- Quite a lot (2)
- Some (3)
- A little (4)
- Not at all (5)

**Q6**

Since the pandemic started, what additional contact have you had with informal local community groups in providing health and/or social care?

- A lot (1)
- Quite a lot (2)
- Some (3)
- A little (4)
- None (5)

**Q7**

Before the COVID pandemic, what contact did you have with the formal voluntary sector in providing health and/or social care (e.g. Age UK)?

- A lot (1)
- Quite a lot (2)
- Some (3)
- A little (4)
- Not at all (5)

**Q8**

Since the pandemic started, what additional contact have you had with the formal voluntary sector in providing health and/or social care (e.g. Age UK)?

- A lot (1)
- Quite a lot (2)
- Some (3)
- A little (4)
- Not at all (5)

**Q9**

If you have been working with the voluntary sector, please explain in what capacity?

|  |
| --- |

**Q10**

How often do you use social prescribing?

- Always (1)
- Most of the time (2)
- Some of the time (3)
- A little (4)
- Not at all (5)

**Q11**

How many organisations deliver social prescribing for you?

|  |
| --- |

**Q12**

Please name the organisations.

|  |
| --- |

**Q13**

What has worked well, with social prescribing since the pandemic?

*Please give examples.*

|  |
| --- |

**Q14**

What has worked less well, with social prescribing since the pandemic?

*Please give examples.*

|  |
| --- |

**Q15**

How do you connect with the voluntary sector?

- Email (1)
- Newsletter (2)
- Telephone (3)
- Word of mouth (4)
- Other (please specify) (5)____________

**Q16**

What benefits have you experienced working with the voluntary sector since the pandemic?

|  |
| --- |

**Q17**

What challenges have you experienced working with the voluntary sector since the pandemic?

|  |
| --- |

**Q18**

Have you had any feedback from patients of the benefits or challenges of receiving services from the voluntary sector?

- Yes (1)
- No (2)

**Q19**

How have you been seeing patients during the COVID pandemic?

|  | Always (1) | Usually (2) | Sometimes (3) | Rarely (4) | Never (5) |
| --- | --- | --- | --- | --- | --- |
| Phone consultation (1) |  |  |  |  |  |
| Video consultation (2) |  |  |  |  |  |
| Face to face (3) |  |  |  |  |  |
| Other (please specify) (4) ____________ |  |  |  |  |  |

**Q20**

In relation to older people, how keen are older patients to engage with digital tools for consultations?

- Always (1)
- Usually (2)
- Sometimes (3)
- Rarely (4)
- Never (5)

**Q21**

Do you have any strategies to encourage them to engage with digital tools?

- Yes (1)
- No (2)

**Q22**

What do you think is necessary for the successful integration of the voluntary sector into Integrated Care Services?

|  |
| --- |

Thank you for taking the time to answer this questionnaire.

**Appendix 2 - Survey for Voluntary and Community Groups**

1. Name of voluntary group:
2. Location:
3. Voluntary group contact details:
4. How many members are there in your group?
5. How did your group form? Please tick one answer:

☐ Spontaneously as a completely new group in response to COVID-19

☐ As a completely new group organised by others (eg Oxford Hub, the local authority, etc) in response to COVID-19

☐ It grew from an existing group (or groups). Please describe what these were:

1. What activities have your group been undertaking to support local people? Please, tick all answers that apply:

☐ Food support (delivery of food)

☐ Donating to food banks

☐ Prescription delivery

☐ Advice and support

☐ Social activities (eg online bookclubs, etc). Please, give details:

☐ Other. Please, give examples:

1. How much of your support activities are connected with NHS health and/or social care provision:

☐ All

☐ Most

☐ About half

☐ Very little

☐ None

1. How much of your support activities are connected with local charities:

☐ All

☐ Most

☐ About half

☐ Very little

☐ None

1. What is the profile of those seeking support in terms of:

Age:

Gender:

Ethnicity:

Nationality:

1. What is the profile of volunteers in terms of:

Age:

Gender:

Ethnicity:

Nationality:

1. What kind of support have members aged over 65 been seeking from the group?
2. What has worked well in your voluntary group?
3. What challenges have you encountered? (Think about the different phases, from setting up your group to delivering support)
4. What communication tools has your group been using to contact members of the community?

Whatsapp

Facebook

Text message

Email

Telephone

Other, please specify.

1. Have you encountered any challenges with any of these tools, in reaching the older population?
2. What tools have the older population used the most, to reach out to your group?

Whatsapp

Facebook

Text message

Email

Telephone

Other, please specify.

1. What do you think is the most effective way of communicating with older people in the community?
2. What resources would help your group to continue supporting your community? (e.g training / funding / people / connections)
3. Are there any relationships with other organisations locally that you have found particularly useful? If so, please provide details.
4. Looking to the future, do you think there are any useful links to formal health and social care services that your group could develop or maintain?
5. Are there any other observations you would like to share?

Thank you for completing the survey.

**Appendix 3 – Survey results from GPs^^[[1]](#footnote-1)^^**

### *1. Context*

The survey was completed by a total of 50 GPs, mainly covering Oxfordshire and the wider South East region. Some 40% of respondents had been qualified for over 20 years, and around half had been in their current position for over 10 years.

When reporting on their contact with the VCSE sector, there was a distinction made between informal community groups (such as local community groups), and more formal voluntary groups (such as Age UK). For more informal community groups, GPs reported having quite a lot of additional contact with informal groups since the start of the pandemic, whereas for more formal voluntary groups, the amount of contact that GPs had before and after the pandemic did not change significantly. Much of the contact with the VCSE sector was through volunteers at Covid-19 vaccinations centres (42%) with other contact through food collection services, delivery of prescriptions and Good Neighbour schemes (befriending), amongst others.

### *2 Social prescription as a link between health and the VCSE sector*

Integrated working through social prescribing was identified as a useful way of connecting with the VCSE sector, with over half (54%) saying they used it some of the time. A further 8% of respondents said they used it most of the time. However, the majority (70%) said they only linked up with one organisation, with a further 18% saying they used two organisations. This suggests that there is considerable scope to enlarge the pool of VCSE organisations that provide social prescribing services to GP practices. Of the organisations mentioned, many (over 30%) were large national bodies (e.g. Age UK, Mind, Marie Curie), with the remaining ones mostly locally-based small-scale voluntary sector organisations.

It was felt by GPs that social prescribing worked well for mental health issues including helping to conquer loneliness (20%), support with other mental health issues and anxiety (8%), as well as keeping people connected and promoting lifestyle changes. Practical benefits included advice on housing, personal finances and benefits, accessing additional care, patient transport, delivery of shopping and prescriptions, and generally keeping connected to the surgery.

However, since the pandemic, it was noted that there has been reduced funding and less resources for organisations offering social prescribing. VCSE organisations have become more difficult to contact, because staff and volunteers have mostly been working from home. Tackling loneliness has become difficult, not only due to the lack of resources for social prescribing organisations, but also due to the lack of face-to-face contact. The volume of need has increased, at a time of reduced services. GPs also reported a lack of awareness of social prescribing services available, the difficulty of bringing organisations together during the pandemic, and a perceived lack of training of the volunteers with appropriate skills.

### *3 Benefits and challenges of working with the VCSE sector*

Many GPs reported on the benefits of working with the voluntary sector. In particular, they cited increased goodwill, with greater community cohesion through helping people to get support at a time when family were unable to help vulnerable members of the community. They became a point of referral and support, helping people with both social and practical aspects of need. The most common benefits reported were support for the isolated elderly, support for mental health and supporting the vaccination centres. GPs felt volunteers were enthusiastic, flexible, helpful, and good team players. They felt that working together improved communication and helped take pressure off GPs, at such a busy time.

However, there were also challenges reported of working with the voluntary sector. As well as reduced funding and staffing of voluntary groups, and the loss of face-to-face interactions, there was also concern around confidentiality and volunteer safety checks. One GP referred to the challenge as ‘crossing boundaries’. GPs also reported concerns around the level of knowledge that the volunteers had, and the need for training. Furthermore, some GPs reported that they were unsure of which voluntary organisation to contact, and felt the voluntary organisations did not promote awareness of their services within healthcare services. This suggests the need for more comprehensive signposting of local VCSE organisations and the services that they provide that could be useful for social prescribing.

### *4 Factors necessary for integration of health with VCSE sector*

GPs were asked to suggest elements that would be necessary for the integration of the voluntary sector into health and social care services. The factors fall into four categories, listed below, as provided by the GPs:

1. Information and communication

- More awareness of what is available and what they can do
- Greater awareness of role
- More information on what’s available and what they can do for us and our patients
- Awareness of need and to match it with available volunteers
- More information about how they work for patients and health-carers
- Discussion between health and social care and the voluntary sector to ensure the right services are offered for the local population
- Better communication and transparency
- Communication and transparency
- Shared vision
- A central signposting service
- A friendly and supportive approach
- Awareness of the benefits.

2. Processes

- Easier access to referral forms/pathways
- Commissioning from the PCN
- Shared protocols/procedures equitable distribution of tasks and responsibilities
- Joined up systems and easier communication pathways
- Formal roles, guidelines and targets
- Continue the current links after the pandemic to benefit the patients
- Ease of access, especially self-referral, both online and print information for patients (e.g. posters/leaflets in waiting rooms)
- More active cooperation with services like social prescribing
- Sound referral pathways
- Confidentiality of information
- Care navigation.
- Be part of the NHS
- Employed by a governing body to do DBS checks.

3. Funding

- Reliable source of funding
- Funding & personnel
- Recruitment & retention of their staff
- Consistent resources and funding
- End user needs analysis
- Money, time and flexibility.

4. Willingness

- Willingness of patients
- Patient education
- Recognising their value and treating them as equal partners
- Willingness to accept change
- Dialogue-ensuring the benefits are highlighted and problems solved - good protocols and training
- Expectation that the roles will change and develop according to the needs of the patient population.

**Appendix 4 – Survey results from the VCSE sector**

### *1 Context*

The survey to the VCSE sector was distributed via the OCVA to their members, as well as to other VCSE stakeholders who had taken part in the research. Despite the low response rate (6 respondents out of a target of 12), some interesting insights have emerged. The six responses represented the different geographies of the study area: two from central Oxford, two from market towns in Oxfordshire and two from rural Oxfordshire villages.

### *2 Pandemic response*

In relation to the profile of those seeking support during the pandemic, most were female and over 55 years old (> 75%), with a mix of ethnicities although mostly white British. In relation to the profile of those providing support (volunteers), most (60%) were over 40 years old, with 25% being over 65 years old. There were many more female volunteers than male (on average, a 65:35 split), with around equal ethnicity of white and non-white.

Many of the services provided related to food shopping and delivery, prescription delivery, dog walking assistance, and phone buddying. The phone buddying scheme that some groups set up was seen as a vital service for older people to have someone to talk to, on a daily basis. Delivering services to those self-isolating was also seen as a way of checking in with those individuals face-to-face, socially-distanced, which went some way to countering issues of isolation and loneliness: *“Food delivery leads to a lengthy doorstep chat with the deliverer, so the food is the catalyst that enables our residents to connect socially.”*

### *3 Partnership working with the health sector*

All but one respondent highlighted the importance of partnerships to their work, collaborating with other VCSE organisations, as well as with the health sector: One commented: *“We work with the NHS eg Oxford Health, GP surgeries, and many local charities”.* Another mentioned working with *“the local advice centre, the social prescriber at the local surgery, all local schools as well as the local Hub team […] This has enabled a great deal of networking and cross referrals to take place and subsequently helping our community”.* There were also other connections to health services, such as the case of one group that is *“currently arranging a series of exercise classes aimed specifically at older people with long term health conditions, so will be working with the surgery to create a referral system.”*

In the words of one respondent:

*‘For a long time now, we have realised that a great deal more can be achieved by working in strong partnership with other agencies and organisations. You create a fantastic pool of skills and because of that, you don’t need to reinvent the wheel, just tap into the resources that are there for you. This way, you share the load and it’s a win-win situation for our targeted audience within our community. One united team is all that is needed to succeed.’*

These examples from the survey illustrate the potential for linking the VCSE sector with health services in partnership at a neighbourhood level, to support the health and wellbeing of local residents.

### *4 Challenges*

A number of challenges were identified by respondents. In particular, reaching out to people who were not on the internet was difficult, especially those who don’t have a wifi connection, smart phones, or who do not use digital devices for communication. In these cases, information about those who needed support was hard to access due to GDPR laws. For those groups that did communicate digitally, Facebook and email were the most common routes. Many groups used leafleting to reach those not connected digitally. In one case, a leaflet and newsletter was hand delivered on a monthly basis to 1250 households.

The sheer volume of demand at the beginning of the pandemic was also a challenge, and which necessitated sign-posting to other sources of support in the area and beyond. There were also concerns, in the case of more formally constituted groups, about funding to support the additional work that was needed, to respond to the surge in demand. *“Funding is by far and large the biggest barrier to us being able to reach out to as many people who would benefit from our support.”*

1. Given the low response rate of pharmacists, with just two completed surveys, their responses have not been included in this analysis. [↑](#footnote-ref-1)
